# Habitat properties are key drivers of *Borrelia burgdorferi* (*s.l*.) prevalence in *Ixodes ricinus* populations of deciduous forest fragments

**DOI:** 10.1186/s13071-017-2590-x

**Published:** 2018-01-08

**Authors:** Steffen Ehrmann, Sanne C. Ruyts, Michael Scherer-Lorenzen, Jürgen Bauhus, Jörg Brunet, Sara A. O. Cousins, Marc Deconchat, Guillaume Decocq, Pieter De Frenne, Pallieter De Smedt, Martin Diekmann, Emilie Gallet-Moron, Stefanie Gärtner, Karin Hansen, Annette Kolb, Jonathan Lenoir, Jessica Lindgren, Tobias Naaf, Taavi Paal, Marcus Panning, Maren Prinz, Alicia Valdés, Kris Verheyen, Monika Wulf, Jaan Liira

**Affiliations:** 1 Geobotany, Faculty of Biology, University of Freiburg, Schänzlestr. 1, 79104 Freiburg, Germany; 2 Forest & Nature Lab, Ghent University, Geraardsbergsesteenweg 267, B-9090 Melle-Gontrode, Belgium; 3 Faculty of Environment and Natural Resources, University of Freiburg, Tennenbacherstr. 4, 79106 Freiburg, Germany; 4 Southern Swedish Forest Research Centre, Swedish University of Agricultural Sciences, Box 49, SE-230 53 Alnarp, Sweden; 5 Landscape Ecology, Department of Geography and Quaternary Geology, Stockholm University, SE-106 91 Stockholm, Sweden; 6 DYNAFOR, Université de Toulouse, INRA, INPT, Chemin de Borde Rouge, CS 52627, F-31326 Castanet, France; 7 UR “Ecologie et Dynamique des Systèmes Anthropisés” (EDYSAN, FRE 3498 CNRS-UPJV), Jules Verne University of Picardie, 1 rue des Louvels, F-80037 Amiens Cedex 1, France; 8 UF PRiMAX, Clinical Pharmacology Department, CHU Amiens-Picardie, Amiens, France; 9 Department of Plant Production, Ghent University, Proefhoevestraat 22, BE-9090 Melle, Belgium; 10 Faculty of Biology/Chemistry (FB 02), Institute of Ecology, Vegetation Ecology and Conservation Biology, University of Bremen, Leobener Str. 5, 28359 Bremen, Germany; 11 Black Forest National Park, Kniebisstraße 67, 77740 Bad Peterstal-Griesbach, Germany; 12 Natural Resources & Environmental Effects, IVL Swedish Environmental Research Institute, Box 210 60, SE-100 31 Stockholm, Sweden; 13 Institute of Land Use Systems, Leibniz-ZALF (e.V.), Eberswalder Str. 84, 15374 Müncheberg, Germany; 14 Institute of Ecology and Earth Sciences, University of Tartu, Lai 40, EE-51005 Tartu, Estonia; 15 Institute of Virology, University Medical Center Freiburg, Hermann-Herder-Strasse 11, 79104 Freiburg, Germany

**Keywords:** Climate gradient, Dilution habitat, Disease ecology, Ecosystem disservice, Functional ecology, Landscape epidemiology, Land-use change, Lyme disease risk, Multi-scale analysis, smallFOREST

## Abstract

**Background:**

The tick *Ixodes ricinus* has considerable impact on the health of humans and other terrestrial animals because it transmits several tick-borne pathogens (TBPs) such as *B. burgdorferi* (*sensu* *lato*), which causes Lyme borreliosis (LB). Small forest patches of agricultural landscapes provide many ecosystem services and also the disservice of LB risk. Biotic interactions and environmental filtering shape tick host communities distinctively between specific regions of Europe, which makes evaluating the dilution effect hypothesis and its influence across various scales challenging. Latitude, macroclimate, landscape and habitat properties drive both hosts and ticks and are comparable metrics across Europe. Therefore, we instead assess these environmental drivers as indicators and determine their respective roles for the prevalence of *B. burgdorferi* in *I. ricinus*.

**Methods:**

We sampled *I. ricinus* and measured environmental properties of macroclimate, landscape and habitat quality of forest patches in agricultural landscapes along a European macroclimatic gradient. We used linear mixed models to determine significant drivers and their relative importance for nymphal and adult *B. burgdorferi* prevalence. We suggest a new prevalence index, which is pool-size independent.

**Results:**

During summer months, our prevalence index varied between 0 and 0.4 per forest patch, indicating a low to moderate disservice. Habitat properties exerted a fourfold larger influence on *B. burgdorferi* prevalence than macroclimate and landscape properties combined. Increasingly available ecotone habitat of focal forest patches diluted and edge density at landscape scale amplified *B. burgdorferi* prevalence. Indicators of habitat attractiveness for tick hosts (food resources and shelter) were the most important predictors within habitat patches. More diverse and abundant macro- and microhabitat had a diluting effect, as it presumably diversifies the niches for tick-hosts and decreases the probability of contact between ticks and their hosts and hence the transmission likelihood.

**Conclusions:**

Diluting effects of more diverse habitat patches would pose another reason to maintain or restore high biodiversity in forest patches of rural landscapes. We suggest classifying habitat patches by their regulating services as dilution and amplification habitat, which predominantly either decrease or increase *B. burgdorferi* prevalence at local and landscape scale and hence LB risk. Particular emphasis on promoting LB-diluting properties should be put on the management of those habitats that are frequently used by humans. In the light of these findings, climate change may be of little concern for LB risk at local scales, but this should be evaluated further.

**Electronic supplementary material:**

The online version of this article (10.1186/s13071-017-2590-x) contains supplementary material, which is available to authorized users.

## Background

Small forest patches, where the ecotone habitat is dominating, are common semi-natural habitats in many European agricultural landscapes [1]. These forest patches play a crucial role in maintaining biodiversity [2] and provide important ecosystem services and disservices (i.e. ecosystem processes which results in benefit or harm for humans) [3, 4]. By providing suitable habitat for the tick *Ixodes ricinus* and many of its host species [5], small forest patches may be an important source habitat of tick-borne pathogens (TBPs), including emerging infectious diseases [6]. In this study we focus on the prevalence of *Borrelia burgdorferi* (*sensu* *lato*) (hereafter labelled *B. burgdorferi*), because it may cause the disease Lyme borreliosis (LB) in humans [7] and is the most commonly reported vector-borne disease of the northern hemisphere [8].

Much research has gone into how the interactions between ticks and their hosts shape the ecosystem disservice of LB risk [9]. The relevant *B. burgdorferi* genospecies have a wide range of vertebrate hosts [10, 11], which are the links to transfer the bacteria from one tick to another. The transmission paths are systemic (and persistent) infection of the host and co-feeding of ticks, where ticks get infected by other nearby feeding ticks, possibly without systemic infection of the host [12]. A weak immune response of the host and a high interaction frequency between host and ticks amplify bacteria transmission between them, and the opposite dilutes it [13, 14]. This defines amplification hosts, which increase the proportion of infected ticks, and dilution hosts, which lower it. More diverse host communities have been shown to dilute *B. burgdorferi* prevalence in North American ecosystems, because they come with a higher density of diluting hosts and hence a higher proportion of blood-meals from hosts that do not disseminate *B. burgdorferi* [15]. However, local host communities vary across the European distribution range of *I. ricinus* due to regionally distinct biotic interactions and environmental filtering [16, 17]*.* This makes predictions about the distribution of *B. burgdorferi* in response to the composition or diversity of host communities across the continental scale challenging.

As both ticks and hosts depend on regional climate, landscape structure and habitat properties, we address various environmental properties simultaneously, which potentially affect both the ticks and the host communities [11, 18, 19]. This enables us to detect drivers of the prevalence of *B. burgdorferi*, which are in contrast to host communities comparable across the European distribution range of *I. ricinus.* We suggest that environmental processes driving the prevalence of *B. burgdorferi* consist of three different conceptual types with underlying scale-dependent processes: (i) tick host driven, (ii) tick abundance driven and (iii) habitat driven.

**Fig. 1:**
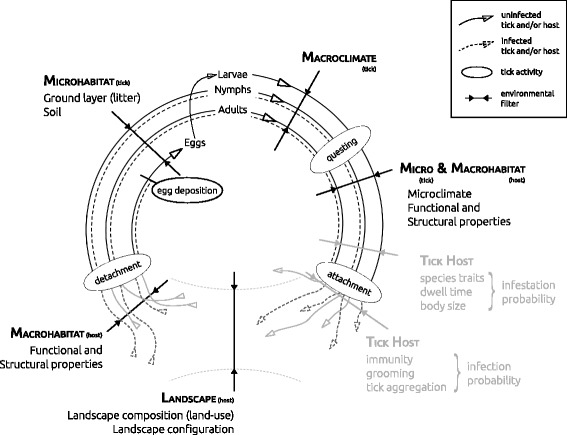
Tick life-cycle with particular emphasis on the driver groups studied here and where they act in the life-cycle. After attachment, ticks are transported with their host. Landscape and habitat characteristics then drive host and tick ecology. Greyed out driver groups (and arrows) are important for the sake of completeness, they are however not included in our analysis. They include not only mechanistic aspects such as ‘tick-host dwell time’ or ‘grooming’, but also molecular effects between ticks, the bacteria and hosts such as ‘tick host species traits’ or ‘immunity’)

(i) Tick host driven: Host species specific infestation [20, 21] and infection probability [13, 22–24], shape the hosts’ competence to acquire and transfer *B. burgdorferi* and hence host dilution/amplification (Fig. 1, greyed out labels). This comprises not only behaviour or traits of the host, but also molecular processes, which are not the topic of this study. Host diversity-pathogen relationships have recently been discussed intensively [15].
(ii) Tick abundance driven: Macroclimate, potentially buffered by the habitat and thus manifesting as microclimate, shapes the temporally and locally-specific abundance of questing ticks (phenology) [5, 25, 26] and with it the synchrony in activity of ontogenetic stages [27]. Larvae often occur clumped on hosts such as small mammals that are at the same time infested by nymphs. In these cases, co-feeding occurs and transmission from nymphs to larvae results in many derived infected nymphs (i.e. infected larvae which immediately after feeding moult into nymphs) [14]. A higher abundance of (potentially infected) nymphs increases the probability of contact between nymphs and hosts, both with or without larval aggregations and therefore with or without co-feeding, hence the likeliness of transmission of *B. burgdorferi* [14, 28]. Due to its mode of action, we label this as ‘tick abundance dilution/amplification’.
(iii) Habitat driven processes can be distinguished between (a) landscape, (b) macro- and (c) microhabitat driven, each of which exert a distinct influence on the prevalence of *B. burgdorferi*. We label these as ‘habitat dilution/amplification’.

- Landscape properties such as the availability and accessibility of habitat suitable for tick hosts may drive tick host density and thus, indirectly, the dynamics shaping *B. burgdorferi* prevalence [29, 30]. For instance, the forest/matrix ecotone drives the density of small mammal and ungulate tick hosts at the landscape scale [31, 32] and this can have cascading effects on tick abundance and *B. burgdorferi* prevalence [33, 34]. Additionally, competition for resources [35] and predation of potential hosts [36] indirectly modify local host communities and may likewise influence the prevalence of *B. burgdorferi.* This is, however, outside the remit of this study.
- Macrohabitat properties shape the *B. burgdorferi* prevalence in questing ticks [22, 37]. This may be due to habitat quality for hosts, in terms of structural (accessibility for shelter and spatial niche distribution) and functional (resources such as dispersules [38]) properties of the habitat patch [16, 29], or due to habitat quality for ticks [5, 25].
- Microhabitat properties (i.e. how the forest understory is spatially arranged) [39, 40] and the microclimate of the understory [41] determine where and when ticks are able to quest. For example, a narrow height distribution of the understory vegetation or a lower diversity may come with less differentiation of niches potentially utilized by ticks and would then bring the questing habitat of ticks spatially closer together [5]. These properties exert distinct influence on the different ontogentic stages (niche differentiation [42]) and shape the stage specific contact between ticks and hosts. Spatio-temporal separation or aggregation of ontogenetic stages presumably shapes the contact probability between the stages and their hosts and influences the transmission of *B. burgdorferi* between them [14, 27]. This also includes contact of distinct ontogenetic stages on the same host, thereby possibly decreasing transmission due to reduced co-feeding [43].

Based on this, we suggest to extend the concept of dilution and amplification from a tick host species to the habitat scale, because both ticks, their hosts, and interactions between them depend upon habitat and landscape features. We define a ‘dilution habitat’ to be a specific habitat, which is generally suitable for tick survival and hampers the transmission of *B. burgdorferi*. Dilution habitats consequently provide a smaller relative amount of infected ticks than other suitable habitats in the surrounding area, which constitutes dilution at landscape scale. In contrast, an ‘amplification habitat’, much like an amplification host, leads to an increased prevalence of *B. burgdorferi* at landscape scale. Both dilution and amplification habitats may depend on one or all of the processes of habitat, tick abundance and tick host dilution/amplification.

We have therefore studied the environmental properties with regard to their capacity as diluting or amplifying factors of the ecosystem disservice of LB risk. We simultaneously estimate the relative role of the driver groups ‘macroclimate’, ‘landscape’, and ‘habitat’ (Fig. 1, Additional file 1: Tables S1-S4) on *B. burgdorferi* prevalence in small forest patches in rural landscapes across temperate Europe. We specifically hypothesize that: (i) extreme macro- and microclimate conditions, resulting in hot and dry summers or cold winters lead to reduced *B. burgdorferi* prevalence; (ii) higher availability and accessibility of forest or forest/matrix ecotone for tick dispersal hosts increases the prevalence of *B. burgdorferi* (an aspect of host dilution); (iii) specific structural and functional properties of the forest patch (macrohabitat) reduce the prevalence of *B. burgdorferi* (an aspect of habitat dilution); (iv) structurally more heterogeneous tick questing habitat (microhabitat) reduces the prevalence of *B. burgdorferi* (an aspect of habitat dilution); and (v) in forest patches with lower questing abundance the *B. burgdorferi* prevalence per respective ontogenetic stage is reduced (tick abundance dilution).

## Methods

### Study locations

**Fig. 2:**
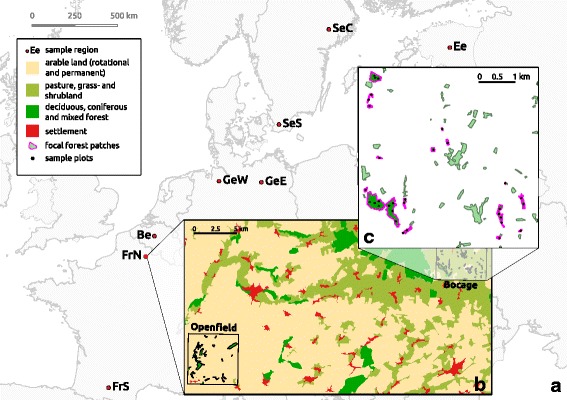
Sampling design of this study. **a** Location of the eight study regions: southern France (FrS), northern France (FrN), Belgium (Be), western Germany (GeW), eastern Germany (GeE), southern Sweden (SeS), central Sweden (SeC) and Estonia (Ee). **b** Detail of a study region depicting the two landscape windows in northern France, showing the most important land-use types and initial deciduous forest patches, the ‘Openfield’-window represents the high intensity land-use and the ‘Bocage’-window the low intensity land-use. **c** Detail of a landscape window depicting a subset of the focal forest patches and sample plots therein

This study was carried out within the framework of the smallFOREST project [44]. Study sites were located in eight regions across the temperate zone of Europe (southern and northern France, Belgium, western and eastern Germany, southern and central Sweden and Estonia, Fig. 2). Two landscape sections of each 5 × 5 km (labelled ‘window’), which contrast in landscape configuration and composition due to differences in land-use intensity, were selected in each of the eight regions. In each of these 16 windows, approximately 16 forest patches of different size and age were selected as focal forest patches. Forest patches had to be dominated by deciduous tree species with more than 60% deciduous cover to be considered for sampling. Our selection resulted in a total of 250 forest patches (Additional file 2: Table S5) throughout Europe. Sampling was confined to predetermined regularly distributed plots in deciduous stands therein (Fig. 2), their number depending largely on patch size (1 to 128 plots per patch, on average 5.0).

The sampling of tick and forest stand characteristics was carried out by the same team in all regions whereas soil and vegetation surveys were performed by site-specific expert groups.

### Ecological surveys

The major setup of our surveys was designed to capture the key drivers as suggested by [18, 19]. We modified it to consider specific properties of rural landscapes and forest as habitat type. We looked at the driver groups ‘macroclimate’, ‘landscape’, and ‘habitat’ and distinguished between macrohabitat (overstory) and microhabitat (understory vegetation, leaf-litter layer and soil) and considered a potential link between ontogenetic stages. Specific indicator traits within each driver group were selected to describe different aspects and to be ecologically meaningful for as many as possible functional guilds related to ticks (such as ticks themselves, potential hosts, plants and leaf litter as habitat). Some meta-variables that are not part of any of these groups were also tested (Additional file 1: Table S1).

#### Tick survey

A random subset of the predefined plots depending on patch size (between 1 and 9 plots per patch, on average 2.3), was sampled for ticks. We collected ticks of all stages of development (larvae, nymphs and adults) by drag sampling [45] in 2013 within 1 week per landscape window (Additional file 2: Table S5) and usually both windows per region consecutively. Due to the high number of plots and their spatial distribution, sampling was possible only once in each plot. Drag sampling was performed with a 1 × 1 m piece of white flannel cloth, attached to a handle. A metal chain was attached at its bottom to increase the contact probability between the cloth and the lower vegetation. Sampling was carried out only during rain-free day time between 09:00 and 21:00 h.

The cloth was dragged upright through the ground-layer vegetation along four 25 m transects in each plot (resulting in a 100 m^2^ sample area). Attached ticks were picked off the cloth after every 25 m and preserved in ethanol. They were counted later in the laboratory and were determined morphologically to species level according to [46]. Small numbers of *Dermacentor* spp. were also encountered, particularly in southern France, but only ticks of the species *I. ricinus* received further consideration. Tick counts per 100 m^2^ were averaged over all plots within one forest patch. Subsequently the averages were log-transformed using the formula x' = log_10_(x + 1).

#### Vegetation survey

The same subset of plots as for the tick sampling was surveyed for forest stand structure, to determine properties of the macrohabitat (Additional file 1: Table S2). For each plot we recorded the tree species, height and number of stems, diameter at breast height (D_130_), the distance and azimuth direction for each tree from the plot centre and whether the tree was dead or alive. Distance and height measurements were performed using a Vertex IV hypsometer (Haglöf Inc., Madison, USA). Sampling was restricted to a 20 m radius from the plot center, to not accidently include a tree in two sample locations, which may coincidently be in close proximity to each other.

Additionally, in all of the predefined plots per patch, plant species composition was surveyed with emphasis on the presence and abundance of all plant species during the 2012 and 2013 growing seasons. These estimations were performed separately for the herb, shrub and tree layers, by assigning one of the abundance categories 1 (< 5 individuals), 2 (5 individuals; 30%) or 3 (> 30%) to each of the present plant species [44].

We derived structural and functional properties at the plot level from the forest stand and vegetation data. To characterize stand structure, we determined stand height, tree density, basal area, tree slenderness coefficients and diameter distributions [47]. To capture structural diversity, we calculated the coefficient of variation (for log-normal data) of the tree diameters and of the potential plant height of the herb layer, the latter derived from the TRY database [48]. The coefficient of variation gives information about the dispersion of the diameters and plant heights and thus how different/diverse they are. Functional traits that are related to the leaf economics spectrum (i.e. traits determining amongst others the decomposition of leaf-litter) [49] (Additional file 1: Table S2) were derived from the vegetation abundance survey using the TRY [48] and LEDA [50] databases. For the herb species, growth and life forms, branching types and specific leaf area were determined (all defined in [50]), because they were assumed to influence the suitability of the herb layer as questing habitat for ticks. Moreover we determined the richness of different weight-classes of dispersules (lightweight: < 0.1 g; medium: 0.1–2 g; heavy: > 2 g, see [38, 50]) and the average overall dispersule mass, separately for all vegetation layers. This served as a proxy for the quality and amount of high energy food that is potentially available for different tick hosts, feeding on these dispersules [51]. Tree leaf traits were weighted by the summed diameter per tree species and all other traits were weighted by the species’ abundance to calculate community-weighted means (CWM) of these traits. These means were then averaged over all plots per patch. Plant species diversity was estimated for the herb-, shrub- and tree-layers as average species richness over all plots per patch. To describe the overall diversity we calculated for each vegetation layer γ-diversity per patch and β-diversity (1-(plot-scale diversity/patch-scale diversity)) as between scale variability [44] (Additional file 1: Table S2).

#### Soil survey

Soil samples were collected between July and October 2012 before leaf fall so that mostly leaves of the previous growth period were part of the leaf litter layer. The subset of plots selected for the soil survey differed slightly from the tick/stand structure and vegetation abundance survey. Soil samples were taken from between 3 and 31 plots per patch (on average 6.0) in accordance to forest patch size. In each plot an area of 25 × 25 cm of the forest floor was sampled according to the method described by [52]. After collecting the forest floor material, the topmost 10 cm of mineral soil was sampled by using a soil corer with a diameter of 4.2 cm. The forest soil layers were analyzed in the laboratory chemically to determine carbon, nitrogen, phosphorous (organic, inorganic and total), ratios and pH (Additional file 1: Table S2).

#### Landscape metrics

We extracted landscape metrics at the patch and landscape scale (Additional file 1: Table S3). At the patch scale we determined the size and age of all forest patches and the proportional area covered by ecotone habitat (buffer of 5, 10 and 20 m into the patch). At the landscape scale, we determined landscape composition in the form of proportion of different land-use types (forest, arable land, pasture) in concentric buffers. Fragmentation is quantified in the form of length per hectare (density) of hedgerow and patch edge, the proximity index and distance of the nearest neighbor forest patch (NND). Additionally we determined the amount of edge habitat inside forest patches at landscape scale (as above) in concentric buffers around focal patches (Additional file 3: Text 1).

#### Climate

Ambient microclimate was recorded at the same time as the tick/stand structure survey with Testo 175-H2 Data-Loggers (Lenzkirch, Germany; temperature precision of ± 0.5 °C, relative humidity accuracy of ± 3%). Measurements were taken every minute for around half an hour in the plot center. Air temperature and relative humidity were measured at a height of both 5 and 130 cm. Soil temperature was measured at a depth of 5 cm. We calculated saturation deficit according to [27], based on values averaged between 5 and 130 cm height for both relative humidity and air temperature.

Macroclimate data were extracted from the Global Summary of the Day (GSOD) dataset hosted on the web-servers of NOAA’s National Centers for Environmental Information (NCEI). We extracted climate metrics for the period from 1st of January 2013 to the day of sampling and for 30 days prior to tick sampling. Additionally, we calculated growing (above 8 °C) and chilling (below 8 °C) degree days from the 1st of January 2013 to the day of sampling. All metrics were averaged over all climate stations within a 20 km radius of the landscape window (mostly two but sometimes only one station was available) (Additional file 1: Table S4).

### *Borrelia burgdorferi* (*s.l*.) prevalence

We pooled ticks per plot, stage and sex to determine the prevalence of *B. burgdorferi* (*s.l*.) in them. Ten nymphs and five male and female ticks were randomly drawn from the overall collected ticks per plot. As adult tick sexes were combined for statistical analyses, the pool size hence varied between one and ten individuals for both stages. The effort of tick collection was planned so that primarily tick abundance would be comparable across all surveyed forest patches. Due to this and the wide geographic gradient, abundance varied from zero to high numbers of around 300 nymphs per 100 m^2^. This resulted in pools of different size, including pools of less than 10 ticks per stage.

In the laboratory, ticks were air-dried and a total of 400 μl minimal essential medium (Gibco™, Waltham, USA) with 0.5% bovine serum albumin (Biochrom AG, Berlin, Germany) and 1% penicillin-streptomycin (Gibco™) was added to each pool. Samples were crushed utilizing Lysing matrix H (MP Biomedicals, Santa Ana, USA) and subsequently centrifuged for 5 min at 3000 g. Of that, 200 μl were subjected to total nucleic acid extraction using the Total NA kit on a MagNA Pure LC 2.0 instrument (Roche, Basel, Switzerland) according to the manufacturer’s instructions. The DNA was eluted to a final volume of μl and samples were stored at -20 °C until PCR analysis. The PCR-procedure described by [53] was slightly modified. The total reaction volume was 25 μl using the FastStart DNA Master HybProbe kit (Roche), using 5 μl of DNA template, 400 nM of each primer, and 110 nM of probe. The cycling conditions on a real-time PCR machine ABI 7500 (Applied Biosystems™, Waltham, USA) were as follows: 95 °C for 10 min followed by 45 cycles of 95 °C for 10 s and 60 °C for 30 s. Negative and positive controls were used throughout. This resulted in a positive or negative *B. burgdorferi* signal for each pool.

Various metrics to estimate infection prevalence in tick pools, such as the infection rate of individual nymphs [54] and the minimum infection rate [55] have been suggested. However, these metrics are biased for ecological inference on the derived pathogen prevalence. Often an inflated sampling effort is required to collect sufficient ticks in less suitable habitat patches, rendering comparability impossible. Therefore, we propose a new index to describe the central tendency of LB risk in a given tick pool, which can be used to compare infection between sites with pools of different size. As prevalence estimates within a pool (percent values) do not add, but multiply together, we used the geometric mean (x̅_p_) of all possible prevalence values, which could occur in a pool of ticks:

1 $$ {\overline{x}}_p={\left({\prod}_{i=1}^{n_p}\frac{i}{n_p}\right)}^{\frac{1}{n_p}} $$

where i is the number of potentially infected ticks per pool p and n_p_ is the number of ticks per pool p.

For instance, given a pool of three ticks, a positive signal may result from one, two or even three infected ticks. The true prevalence of this pool may thus be 1/3, 2/3 or 1 and its geometric mean of prevalence probabilities is 0.61. The value range of this metric is, like the true prevalence, bounded by 0 and 1. However, while this metric is comparable across studies, it is not comparable to other measures of prevalence, as it leads to higher estimates than with other methods [56].

To derive the patch average all plots per patch were averaged:

2 $$ \overline{X}=\frac{1}{N}{\sum}_{p=1}^N{\overline{x}}_p $$

where x̅_p_ is the geometric mean of all potential probabilities of pool p and N is the total number of pools per patch.

By sampling only a subset of ticks we inevitably introduce a detection threshold to LB prevalence. The more ticks we sample per patch, the more sure we can be that a negative signal is a true absence of the infection. To account for this, we calculated theoretical values of infection prevalence for patches, in which ticks have been found, but an infection has not been detected in the laboratory:

3 $$ \overline{X}=\frac{{\overline{x}}_{pmin}\cdot 0.9}{\sum_{p=1}^N{n}_p} $$

where x̅_p min_ is the smallest possible prevalence value (≠ 0) calculated with Eq. 1 (i.e. mathematical detection threshold) and n_p_ is the number of ticks per pool p and N = total number of pools per patch.

### Statistical analyses

All statistical work was carried out in R version 3.3.1 [57]. An explanatory factor analysis (EFA) was carried out, based on maximum likelihood, to derive factors of correlating variables (Additional file 4: Text 2, Additional file 5: Table S6) within variable groups, with the ‘psych’ package [58]. These correlation factors are assumed to represent the combined (and more general) influence of a set of correlating variables, which would not be significant separately because they might be too specific. The factors were then used in the same way as the other drivers for model building.

**Fig. 3:**
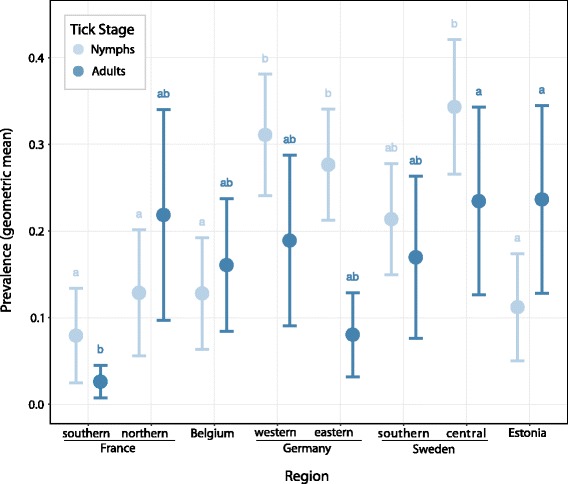
Average prevalence of *Borrelia burgdorferi* (*s.l*.) per region. The whiskers represent the 95% confidence interval. Whiskers with the same letter do not differ significantly (Tukey’s test)

We derived the letters indicating differences between patch averages per region (Fig. 3) with the multcompView package [59].

We built linear mixed models (LMMs) to explore the effect of all environmental variables on the *B. burgdorferi* prevalence in *I. ricinus* ticks using the *lme4* package [60]. We transformed prevalence with the logit function (after adding 0.009 to each probability, to avoid infinite values in case of zero probabilities) and fitted an LMM to these values for nymphs and adults (larvae are usually free of *B. burgdorferi*). We implemented ‘region’ as random factor (eight regions = eight levels) to account for variation in the response to regional differences such as in seasonality (i.e. distinct development of phenological events), specific land-use and faunistic composition.

We developed a standardized, semi-automatic variable selection procedure, including second order polynomials for effects, as described in Additional file 6: Text 3 and Additional file 7: Figure S1. The derived models were eventually fit using restricted maximum likelihood (REML). The *lmerTest* package [61] was used to determine type-III ANOVA tables with the Wald F-test with Satterthwaite degrees of freedom for fixed effects. Response profiles based on partial residuals were determined with the *visreg* package [62] and plotted with *ggplot* [63]. We derived the relative importance of the selected drivers based on partial eta^2^ values (Additional file 8: Text 4) [64]. Detailed R-code, which covers model building and preparation of graphs can be found on github (https://github.com/EhrmannS/2017_Parasit-Vectors_Habitat-properties-are-key-drivers-of).

## Results

### Distribution of ticks and *B. burgdorferi* (*s.l*.)

We analysed 4146 ticks and 77.6% (3218) thereof were nymphs (please see Additional file 2: Table S5 for sampling location and dates). An average from 3.4 (southern France) to 26.3 (eastern Germany) nymphs were analysed per patch. Since fewer adults were found, only between 0.9 (southern France) and 7.6 (Belgium) adults (both sexes combined) were tested per patch. The average geometric mean of *B. burgdorferi* prevalence per patch as a proxy for infection prevalence ranged for nymphs on average from 0.08 and 0.11 in southern France and Estonia, up to 0.31 and 0.34 in western Germany and central Sweden. For adults, these values ranged from 0.03 in southern France up to 0.23 and 0.24 in central Sweden and Estonia (Fig. 3).

**Fig. 4:**
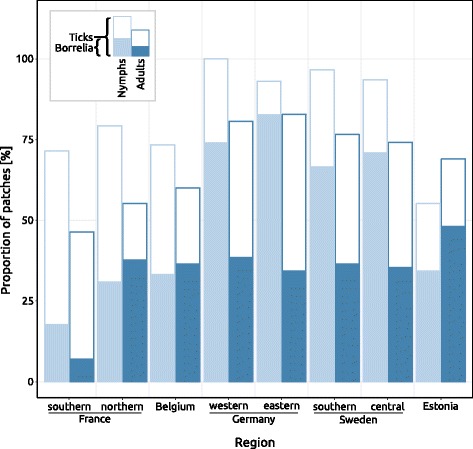
Fraction of patches with ticks and Lyme borreliosis along the latitudinal gradient

We found nymphs in 55% of forest patches in Estonia and in nearly all patches in southern Sweden and western Germany (97 and 100% respectively). The lowest fraction of patches with infected nymphs however, was found in southern France (18%) and the highest fraction in eastern Germany (83%) (Fig. 4). We found infected nymphs in less than 50% of the forest patches also in northern France, Belgium and Estonia.

Adults were generally found in fewer patches than nymphs, from 46% of patches in southern France to 83% of patches in eastern Germany. The lowest fraction of patches with infected adults was found in southern France, where only 7% were infected, and the highest fraction was found in Estonia (48%) (Fig. 4).

### Model results

The final parsimonious models explained the variation of infection prevalence with *R*^2^_adj_ = 0.55 for both nymphs and adults. We found a wide range of significant environmental variables, combined with a large effect on the prevalence of *B. burgdorferi* in nymphs (Additional file 9: Table S7) and adults (Additional file 9: Table S8).

**Fig. 5:**
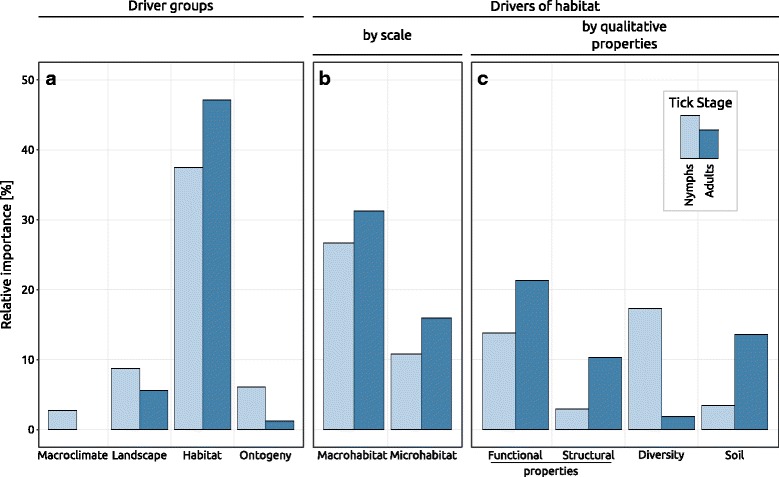
**a** Relative importance of categories of drivers in percent. Within ‘habitat’, drivers were grouped either according to **b** scale within habitat or according to **c** further sub groups. Both, the bars in **b** and in **c** add up to the relative importance of ‘habitat’ in **a**. Variables were grouped according to Additional file 1. ‘Diversity’ is composed of functional and structural, but also taxonomic (i.e. species based) diversity. Relative importance is the relative contribution of all η^2^ values of a group to the overall variation in the tick abundance data related to the fixed-effects part of the models

Variables describing macroclimate conditions explained only a negligible part of variation. Merely the number of days above 8 °C between the 1st of January and the day of sampling was significant for nymphs and had a negative effect with a relative importance of 2.7% (Fig. 5). Variables of ‘macroclimate’ were not identified as significant for adults.

Variables describing landscape comprised the second most important driver group (explaining 8.7 and 5.5% of variation in infection prevalence for nymphs and adults, respectively). The proportion of agriculture in the adjacent landscape (100 m buffer) was the most important variable (4.4%) and the proportion of edge habitat explained another 0.7% of *B. burgdorferi* prevalence in nymphs. *Borrelia burgdorferi* in nymphs was explained by drivers in relation to fragmentation, namely edge and patch density (1.8 and 1.0%, respectively) and by the proximity of other forest patches (0.9%). The prevalence in adults was explained by very similar landscape drivers, namely the factor combining variables of the proportion of edge habitat (all scales) (3.1%) and the proportion of agriculture on a wider landscape context (1000 m buffer, 2.4%).

Variables describing habitat properties accounted for the largest part of variation of nymphal (37.5%) and adult *B. burgdorferi* prevalence (48.0%). Microclimate, as part of habitat properties, neither explained variation for *B. burgdorferi* in nymphs nor in adults. For both stages ‘macrohabitat’ was more important than ‘microhabitat’ (Fig. 5b). The *B. burgdorferi* prevalence in nymphs was explained less by macro- and microhabitat (26.7 and 10.8%) than the *B. burgdorferi* prevalence in adults (31.2 and 15.9%). Similarly, functional, structural and soil properties were less important for *B. burgdorferi* in nymphs (13.8, 2.9 and 3.4%, respectively), than in adults (21.3, 10.3 and 13.6%, respectively). However, the diversity of various vegetation strata was a more important predictor for *B. burgdorferi* in nymphs (17.3%) than in adults (1.9%) (Fig. 5c). The most important single drivers of nymphal *B. burgdorferi* prevalence associated with ‘macrohabitat’ were the abundance of shrubs with nuts (4.4%) with a negative effect, and the beta-diversity (4.1%) and the proportion of *Prunus* spp. in the patch (3.8%), both with a positive effect on infection prevalence. The most important ‘microhabitat’ drivers for nymphal infection prevalence were the richness of saplings (5.6%), the abundance of evergreens (*Hedera helix*, *Vinca* spp.) (2.6%) in the herb layer and the amount of large deadwood (1.4%), all with a negative effect (Additional file 10: Figure S2). For *B. burgdorferi* prevalence in adult ticks the most important single drivers associated with ‘macrohabitat’ were the abundance of shrubs with dispersules > 0.1 g (6.3%) and the specific leaf area of trees (3.8%) with a considerable negative effect on infection prevalence. In contrast, the richness of lightweight dispersules (< 0.1 g) in the tree layer (4.8%) had a positive effect on the *B. burgdorferi* prevalence of adults. Several other ‘macrohabitat’ drivers had a moderate to small (< 2%) effect (Additional file 11: Figure S3). Among ‘microhabitat’ drivers, the specific leaf area of the leaf-litter had a considerable non-linear effect with pessimum (i.e. u-shaped) (5%) and the abundance of the herb layer (3.7%) and of deadwood (1.2%) had a negative effect on adult infection prevalence (see Additional file 1: Tables S1-S4 for the exact association of variables to driver groups).

Both nymphal and adult infection prevalence correlated positively with the abundance of each respective stage, which explained 6.1% of infection prevalence in nymphs and 1.2% in adult ticks. (Fig. 5).

## Discussion

We have shown that the prevalence of *B. burgdorferi* in nymphs and adults is driven to a large degree by habitat level drivers, in terms of functional and structural properties and by landscape, in terms of habitat fragmentation and landscape composition (land-use). Habitat dilution is hence a clear and important driving force of *B. burgdorferi* prevalence of ticks dwelling in deciduous forest patches of agricultural landscapes. Effects of macroclimate and ontogeny were the least important and expressed mostly for nymphs.

### Macroclimate

Macroclimate did not explain considerable variation in *B. burgdorferi* prevalence (Fig. 5). Neither latitude, longitude or widely used mean temperatures of the season, provided any explanatory power for cross-regional variability. Instead, only the number of days above 8 °C between the 1st of January and the day of sampling, which reflects the winter mildness and the sampling date, was significant. It is important to recall at this point that we did not sample all regions throughout the year (and hence do not provide a full phenological analysis) but that we sampled forest patches of one region within a relatively short timeframe once per year. We were not able to fully synchronize the day of sampling for all regions so that we would have sampled ticks precisely in the same phenological phase across the gradient. The resulting different seasonality of our sampling campaign is expressed through the specific manifested macroclimate and the phenological phase. However, seasonality varies between regions and years [65–68], as does the length of the growing season. For instance, in Estonia the number of warm days may be smaller than in eastern Germany, even though Estonia has been sampled later in the year and this is because the phenological development differs along latitude [66, 69]. Both non-synchronised sampling and regional differences in macroclimate enabled us to statistically explore variation in the response variable also with respect to macroclimate (phenology), albeit not on an as strong gradient as for landscape and habitat properties.

Due to the confoundedness of winter mildness and sampling date, we were not able to unambiguously identify the process behind the diluting effect of the significant macroclimatic metric. Two interpretations may be possible. A higher number of warm days indicates a longer growing season and may shape microhabitat composition and hence the contact possibility between ticks and hosts and tick stages on hosts. Alternatively, it may indicate asynchronised questing activity of the different tick stages [27]. This would be based on the assumption that macroclimate limits the questing abundance of ticks throughout the year, and hence influences B. burgdorferi prevalence via increased or decreased manifested contact of separately questing tick stages [70]. However, these two interpretations are not mutually exclusive. In conclusion, macroclimate seems to influence the transmission of B. burgdorferi by driving certain environmental processes in forested habitat patches and indirectly host-tick contact, partly confirming hypothesis (i).

### Landscape

In rural landscapes of Europe, the majority of remaining forest is no more than 100–200 m away from the forest edge [1]. The resulting high edge density at landscape scale has previously been suggested to lead to a higher *B. burgdorferi* prevalence [33, 71]. Edge density is a proxy for ecotone habitat at landscape scale, which is suitable for tick hosts such as roe deer [32], as it increases their abundance and mobility. These hosts disperse large amounts of ticks where they dwell and thereby increase the local tick-to-host ratio. Moreover, a closer proximity of neighbouring forest patches and decreasing patch density had an amplifying effect on nymphal infection prevalence. Together, these metrics indicate increasing forest cover, which is split up into less distinct patches (i.e. more connected), in turn increasing the local abundance of ticks [31, 34].

However, the effect of increasing edge density has previously been interpreted as increasing the amount of ecotone area within a forest patch, which would have shifted small mammal communities towards higher density of edge-inhabiting species. A high density of highly *B. burgdorferi* competent edge-dwelling hosts, such as the wood mouse (*Apodemus sylvaticus*) [23], was then suspected to be the reason for *B. burgdorferi* amplification here [33]. Since we instead found that the proportion of ecotone of the focal forest patch (note: we distinguish between patch and landscape scale here) had a diluting effect on *B. burgdorferi* prevalence, we cannot readily attribute this effect to higher density of possibly amplifying hosts. An alternative interpretation may be based on dilution due to a reduced tick-to-host ratio. While *A. sylvaticus* may in fact be dominating in many wooded ecotone habitats [31, 72], the overall abundance of small mammals also increases with ecotone availability [72, 73], and the land-use type determines context specific small mammal communities [74]. Generally increasing small mammal density leads to a lower tick-to-host ratio, given the tick abundance is controlled for, with a stochastic effect on *B. burgdorferi* transmission events [28, 73]. Consequently, this tick-to-host ratio interpretation can also explain the amplifying effect of higher tick loads where ungulate tick-hosts dwell.

We conclude that effects of landscape configuration and composition likely drive animal occurrence and mobility, thereby shaping the local tick-to-host ratio with cascading effects on *B. burgdorferi* transmission. Our results hence support hypothesis (ii), albeit the combined relative importance of all landscapes effects is only moderate in our landscape context (Fig. 5).

### Tick host habitat (macrohabitat)

Denser forest stands (i.e. higher tree density, higher basal area) had an amplifying effect on *B. burgdorferi* prevalence. This could be due to the lower preference or reduced accessibility of such stands for ungulate tick hosts [75], which could otherwise act as dilution hosts. Alternatively, an increased microclimatic buffering potential of denser forest stands [76] exposes ticks to less extreme microclimatic conditions and comes with higher survival of ticks.

Higher structural and functional diversity (richness of large trees, diameter diversity, richness of dispersules or amount of deadwood) had a diluting effect on *B. burgdorferi* prevalence. More diverse stands may provide more differentiated niches, thus allowing for a more diverse community of tick hosts, with diluting effects on *B. burgdorferi* prevalence [15]. However, contrasting results of tree diversity in European forest patches on *B. burgdorferi* prevalence exist [77].

Hosts are affected by the functional quality of the stand [16]. For instance, we have shown that food sources for small mammals [78], roe deer and wild boar [79] or birds [80] are important drivers for both, nymphal and adult infection prevalence. Food sources were not limited to acorns [81], but encompass additional species of the genera *Corylus*, *Fagus*, *Frangula*, *Prunus* and *Sorbus*, with intermediate dispersule mass. The effects of dispersules from *Sorbus* and *Prunus* suggest that birds play a role in *B. burgdorferi* prevalence dynamics [82] also in the forest patches we studied. Dispersules may be particularly important in and after mast years [35], when they increase small mammal and other tick host densities leading to lower tick-to-host ratio and hence presumably *B. burgdorferi* prevalence [28].

We conclude that hypothesis (iii) was supported by our results, as we found a diluting effect of good habitat quality for tick hosts and amplifying effects of less suitable conditions. We suggest that the role of dispersules on host community composition should be studied more in detail. Generalist feeding behaviour [83, 84] and biotic interactions between guilds of post-dispersal seed predators [17] allow for context specific adaption of tick host communities, in response to variable food resources [51]. The relative importance of these drivers in our study shows, nevertheless, that the quality of tick host habitat plays a crucial role in driving *B. burgdorferi* prevalence in small forest patches of agricultural landscapes.

### Tick dwelling habitat (microhabitat)

For both nymphal and adult infection prevalence, higher herb layer density had a diluting effect. Understory vegetation and particularly the herb layer are questing habitat for ticks [27, 39] and small mammals are often more abundant with higher vegetation cover [35, 85]. Both aspects combined increase the likelihood of ticks finding a host, but decrease at the same time the likelihood that many ticks will make contact with the same host. This would lower the average tick burden and with it the co-feeding probability of different tick stages on small mammal hosts, which may explain the dilution of *B. burgdorferi* prevalence [28].

The specific vertical structure of the herb layer seemed to have influenced the *B. burgdorferi* prevalence in nymphs, but not in adults. Ticks adapt their questing height in response to the microclimate [27, 39], we did, however, not find an effect of a single microclimatic metric on the *B. burgdorferi* prevalence in nymphs and adults. This indicates that microhabitat structure may overrule microclimate. If the ticks’ questing habitat is restricted because it is dominated by plants with a compact growth form, such as chamaephytes, which often grow close to the ground [86], nymphs are restricted to quest close to the ground, even under for ticks’ ideal microclimatic conditions. In contrast, taller herb layer plants with regularly distributed leaves, as an interface for questing ticks, allow for a more spatially differentiated questing activity. This reflects the probability of tick feeding on different host individuals or species and thus the dilution/amplification capacity of the understory vegetation.

The diversity and functional composition of tree species [87, 88], forest floor decomposer communities [87, 89] and also forest management practices [90], can potentially drive leaf-litter decay. Indeed, humid soils with a well-developed leaf-litter layer have been shown to support higher densities of ticks [22, 37, 91], but an influence on *B. burgdorferi* has hardly been shown. We can, therefore, only assume that the significant variables of the leaf economic spectrum or leaf litter SLA, which are proxies of leaf-litter decomposition [92] may drive *B. burgdorferi* prevalence via the availability of tick microhabitat within the litter [5]. For instance, *Quercus* spp. and *Fagus* spp. have slowly decomposing leaf litter, forming a well-developed forest floor, which supports the survival of ticks [22, 37]. Under adverse microclimatic conditions nymphs and adults also dwell in the forest floor, if it is available [27], resulting in an overlap of tick stages. Similarly, as with understory vegetation, the abundance of the leaf litter shapes the contact probability of ticks with hosts and other ticks, presumably with diluting/amplifying effects on *B. burgdorferi* transmission [27].

To conclude, while controlling for tick abundance, habitat dilution due to structurally more heterogeneous tick questing habitat (microhabitat) is likely, supporting hypothesis (iv). However, not only a more diverse habitat but also denser vegetation seems to have a diluting effect.

### Ontogeny

We found an amplifying effect of the abundance of both nymphs and adults on their respective *B. burgdorferi* prevalence. However, their explanatory value was low, which indicates that habitat and landscape conditions are more important drivers of *B. burgdorferi* prevalence, than tick abundance itself. Yet, in conclusion, this result supports the tick abundance amplification assumption [28], that a higher abundance of each tick stage leads to higher *B. burgdorferi* prevalence.

### Dilution of an ecosystem disservice

We have shown that the landscape matrix and environmental properties of forest patches have to be considered as important drivers of *B. burgdorferi* prevalence. We found many habitat quality related effects, which we suspect to shape the contact between ticks and hosts and ticks on hosts. They implicate that a lower tick-to-host ratio may eventually be responsible for dilution of *B. burgdorferi* prevalence at the habitat scale. We have identified dilution habitats to have diverse and abundant understory vegetation and increased levels of deadwood, yet low beta-diversity and low availability of, for instance, *Prunus* in the stand. This strong link between the prevalence of *B. burgdorferi* and habitat properties, which can potentially be managed by humans, emphasizes the responsibility of forest and landscape managers for LB risk. In practice, land-use change and habitat degradation seem to overrule the effects of climate change on LB prevalence.

It has to be noted that in regions where the abundance of ticks is generally high, even moderate or low *B. burgdorferi* prevalence may be indicative of a considerable abundance of infected ticks. This implies that what has been identified as a dilution habitat in one region, may not necessarily also be a dilution habitat in another region. However, in a region with high tick pressure, a dilution habitat also provides a lower *B. burgdorferi* prevalence, than surrounding habitat patches relative to a regional average level.

## Conclusions

Extending the meaning of “diluting the pathogen prevalence in ticks” from tick-host species to the habitat and landscape scale may help us to better understand the ecological dynamics of TBPs. The concept of “dilution habitats”, in contrast to merely “dilution hosts”, should thus find consideration in future works to improve our understanding of the cycling of *B. burgdorferi* at landscape scale. For adapted ecosystem management, we have to identify habitat patches, including other habitat types such as coniferous forest and (semi-natural) grasslands, or even complete landscapes, which consistently have diluting properties. Humans should be in the focus of attention when dealing with ecosystem (dis)services, such as LB prevalence. Dilution habitats alleviate the effects of this important disservice directly at local scale, where it is relevant for humans, but also at regional scale. The latter aspect manifests in dilution habitats in a reduced burden of infected ticks for important hosts, which dwell in the vicinity of humans and which disperse ticks in the landscape (i.e. ungulates and birds). The concept of habitat dilution, in addition to host dilution, is a promising concept to understand landscape epidemiological processes and translate the vast amount of already given research into action to lower health related risk of LB and other TBPs.

## Additional files

Additional file 1: Focal variables as potential environmental drivers of *B. burgdorferi* prevalence. **Table S1.** Meta-variables. **Table S2.** Variables of habitat. **Table S3.** Variables of landscape. **Table S4.** Variables of macroclimate. (XLSX 40 kb)

Additional file 2: Table S5. Meta-data on study setup. Outline of the most important meta-data, such as center of each studied landscape window, dates of sampling, number of forest patches and a couple of landscape metrics to characterize the respective landscapes. (XLSX 26 kb)

Additional file 3: Text 1. Methods: technical details. (DOCX 16 kb)

Additional file 4: Text 2. Details on how correlation factors were derived and what the name components of the internal variables mean. (DOCX 15 kb)

Additional file 5: Table S6. Loadings of all correlation factors. (XLSX 16 kb)

Additional file 6: Text 3. Details on the model building/variable selection procedure. (DOCX 12 kb)

Additional file 7: Figure S1. Flow chart of the overall data processing procedure. (PDF 46 kb)

Additional file 8: Text 4. Equations according to which effect sizes for significant drivers were calculated. (DOCX 6 kb)

Additional file 9: Model output **Table S7.** Nymphal infection prevalence. **Table S8.** Adult infection prevalence. (XLSX 18 kb)

Additional file 10: Figure S2. Response profiles of infection prevalence for each significant driver. Shown are the prediction line, confidence band (alpha = 0.05) and the partial residuals. The driver groups are macroclimate, landscape, macrohabitat, microhabitat and ontogeny. η^2^ values represent the relative contribution each variable has in explaining variation in the response. Nymphal infection prevalence. (FA) = variable is a correlation factor, ‘abund.’ = abundance, ‘disp.’ = dispersules, ‘cont.’ = content, CWM = community weighted mean, ‘reg. Leaf-dist.’ = leaf distribution regular on stem. (PDF 332 kb)

Additional file 11: Figure S3. Response profiles of infection prevalence for each significant driver. Shown are the prediction line, confidence band (alpha = 0.05) and the partial residuals. The driver groups are macroclimate, landscape, macrohabitat, microhabitat and ontogeny. η^2^ values represent the relative contribution each variable has in explaining variation in the response. Adult infection prevalence. (FA) = variable is a correlation factor, ‘abund.’ = abundance, ‘disp.’ = dispersules, ‘SLA’ = specific leaf area, ‘tree2’ = lower tree layer. (PDF 254 kb)
