# Supplementary material for: Habitat properties are key drivers of Borrelia burgdorferi (s.l.) prevalence in Ixodes ricinus populations of deciduous forest fragments
Source: Parasit Vectors. 2018 Jan 8;11:23. doi: 10.1186/s13071-017-2590-x (PMC5759830; doi:10.1186/s13071-017-2590-x)
Supplement: Supplementary file 3 — Methods: technical details. (DOCX 16 kb) [file 13071_2017_2590_MOESM3_ESM.docx]

1. **Additional file 3: Text 1.**

## Methods – technical details

### Tick survey

After sampling, the wetness of the cloth was estimated with the categories 'dry' (74.0% of drags), 'bottom moist' (10.4%), 'moist' (13.0%), 'bottom wet' (0.5%) and 'wet' (2.1%). The category 'moist' was assigned when the first signs of water (e.g. drops from the vegetation) were perceptible on the flag. As all values were collected on plot level and subsequently averaged at patch level, also the wetness of the flag was treated like that. We assigned numerical values to each of the categories and averaged these over patches. After this, 77 % of patches (190) had an average value of 1, which was assigned to a dry flag. Another 20.6 % of patches (51) had a value larger than 1 and smaller than 3, which were assigned to flags, which were at the bottom or overall moist. The remaining 2.4 % of patches (6) were sampled with a wet or partially wet flag. Of these, 5 patches were in southern France. We are aware that averaging of categorical values is strictly speaking not allowed, however, since these averages reflect quite well the plot-level values, we are confident that model results are hardly more unreliable due to this. Non-integer average values indicate that a patch was sampled with a different flag condition in its plots, but these cases are relatively rare (7.5 %).

Sampling of ticks occurred within maximum one week with only one exception. In Belgium heavy rains set in shortly after sampling started and we hence moved to another region and continued sampling of the Belgian landscape windows about a month later.

### GIS work

Landscapes were characterized utilizing ArcGIS 10.3 (ESRI 2014), based on maps acquired within the framework of smallFOREST [1]. Maps were digitized from recent aerial photographs and historical maps of the last centuries. Buffers for land-use metrics were 50, 100, 250, 500, 1000 and 5000 m.

The age of each recent patch was calculated as specified in [1]. It was based on the first appearance of the patch in historic maps. However, since a recent forest patch may be composed of sub-patches, which appeared at different times throughout the last centuries, the patch age was consequently calculated as area-weighted mean of the age of all sub-patches.

### Forest stand characterisation

The Point-Center-Quarter (PCQ) method [2] was used to select trees for measuring stand structure. Two trees per quarter were measured, so that they would be of small and large diameter to represent different layers or age classes. Small trees were chosen to be between 7 cm and 30 cm in diameter at breast height (D_130_) and large trees were larger than 30 cm at D_130_. In addition to the PCQ survey, four neighbouring trees were measured (“structural group of four” [3]). The tree closest to the plot center was chosen as reference tree and the same set of variables, except tree height were determined.

### Soil Analysis

Bulk density was estimated from soil samples and used to quantify the pools of carbon and nitrogen after chemical analysis. The samples were dried at 40 °C and weighed. The soil was passed through a 1 mm sieve to remove stones and gravel. Soil pH (CaCl_2_) was measured using a glass electrode (Orion, Orion Europe, Cambridge, England, model 920A) following the procedure described in [4]. The concentration of carbon and nitrogen was measured by high temperature combustion at 1150°C using an elemental analyser (Vario MACRO cube CNS, Elementar, Germany). After complete destruction with HClO_4_ (65%), HNO_3_ (70%) and H_2_SO_4_ (98%) in Teflon bombs for four hours at 150 °C total phosphorous concentrations in mineral soil were measured according to the malachite green procedure [5].

### Microclimate

For analysis, air temperature at 5 cm and 130 cm height were averaged, since they correlated highly (r = 0.92) and did not differ significantly (t-test, p = 0.5). Relative humidity values were also correlated (r = 0.75), but did differ between 5 cm and 130 cm height (t-test, p < 0.001) and were thus treated separately.

## References

1. Valdés A, Lenoir J, Gallet-Moron E, Andrieu E, Brunet J, Chabrerie O, et al. The contribution of patch-scale conditions is greater than that of macroclimate in explaining local plant diversity in fragmented forests across Europe: Drivers of herbaceous species diversity in fragmented forests. Global Ecology and Biogeography. 2015;24:1094–105.

2. Cottam G, Curtis JT. The Use of Distance Measures in Phytosociological Sampling. Ecology. 1956;37:451–460.

3. Pommerening A. Approaches to quantifying forest structures. Forestry. 2002;75:305–324.

4. Chemical methods and soil characteristics I 190/SC 3. ISO 10390:1994(E) [Internet]. 1994. Available from: https://www.iso.org/standard/18454.html

5. Lajtha K, Driscoll CT, Jarrell WM, Elliott ET. Soil phosphorus. Characterization and total element analysis. In: Robertson GP, Coleman DC, Bledsoe CS, Sollins P, editors. Standard soil methods for long-term ecological research. Oxford University Press; 1999. p. 115–42.
