# Supplementary material for: Habitat properties are key drivers of Borrelia burgdorferi (s.l.) prevalence in Ixodes ricinus populations of deciduous forest fragments
Source: Parasit Vectors. 2018 Jan 8;11:23. doi: 10.1186/s13071-017-2590-x (PMC5759830; doi:10.1186/s13071-017-2590-x)
Supplement: Supplementary file 4 — Details on how correlation factors were derived and what the name components of the internal variables mean. (DOCX 15 kb) [file 13071_2017_2590_MOESM4_ESM.docx]

**Additional file 4: Text 2.**

## Correlation factors

Several data frames were extracted from the overall data frame. The code for this is omitted, since it comprises only selection of the relevant columns. These data frames are macroclimate, landscape, structure, traits, taxonomic, abundance, soil, microclimate. The factor analysis was carried out with psych::fa() and was based on maximum likelihood factoring, scores were found using regression and the number of factors to extract was determined with the function psych::fa.parallel() without any further arguments specified. Explanation for variable names can be found in Tab. A4.1.

| Tab. A4.1: meaning of name components of the here used variables. |
| --- |
| \| **name component** \| **meaning** \| \| --- \| --- \| \| 50 – 5000 \| for the buffer of respective size \| \| abund \| abundance \| \| all \| for plants in all layers \| \| alpha \| alpha diversity (richness) \| \| asc_to_pros \| plants with ascending or prostrating habitus \| \| asin \| arcsin transformed \| \| ba \| basal area \| \| beta \| beta diversity \| \| bitterlich \| according to the bitterlich sampling \| \| bn \| binary, is the property available or not? \| \| c.cont \| carbon content \| \| c.n \| C/N-ratio \| \| canopy height \| potential canopy height of plant layer \| \| cdd \| chilling degree days \| \| cult \| cultivated, agricultural land-use \| \| cv \| coefficient of variation \| \| dead \| dead standing trees \| \| decid \| for deciduous plants \| \| diam \| diameter \| \| diff \| differentiation \| \| disp \| dispersule \| \| diss \| dissimilarity \| \| dw \| deadwood \| \| edge5, 10, 20 \| of the first 5, 10, 20 m inside the patch edge \| \| evergr or e \| (also) for evergreen plants \| \| ff \| forest floor \| \| gamma \| gamma diversity \| \| gdd \| growing degree days \| \| hedg \| hedgerow \| \| height_stand \| height of crown trees \| \| herb \| for herb layer plants \| \| inorg \| inorganic \| \| iqr \| interquartile range \| \| l1mm_mat \| material larger than 1 mm in diameter (soil) \| \| large (in combination with 'disp') \| dispersule > 2000 mg mass \| \| large (in combination with 'tree') \| for trees with d130 > 30 cm \| \| lg \| log transformed, usually before calculating patch average \| \| max \| maximum value of all plots per patch \| \| md \| minimum distance between all trees of one plot \| \| mean \| average over all plots per patch \| \| med \| median \| \| medium \| dispersule 100 mg < x < 2000 mg mass \| \| medium_large \| dispersule > 100 mg mass \| \| min \| minimum value of all plots per patch \| \| ms \| mineral soil \| \| n \| number of something \| \| n.cont \| nitrogen content \| \| n.p \| N/P-ratio \| \| NND \| nearest neighbour distance \| \| org \| organic \| \| p.cont \| phosphor content \| \| past \| pasture \| \| pre \| 30 days previous of sampling \| \| precip \| precipitation \| \| PROX \| proximity index \| \| rH \| realtive humidity \| \| rich \| alpha diversity (richness) \| \| satdef \| saturation or vapour pressure deficit \| \| short \| trees in the lower 50% of the size-values of the plot \| \| shrub \| for shrub layer plants \| \| sla \| specific leaf area \| \| small (in combination with 'disp') \| dispersule < 100 mg mass \| \| small (in combination with 'tree') \| for trees with d130 < 30 cm \| \| spl \| saplings \| \| sqrt \| square root transformed \| \| tall \| trees in the upper 50% of the size-values of the plot \| \| temp \| temperature \| \| tree \| for tree layer plants \| \| tree1, tree2 \| upper, lower tree layer \| \| vol \| volume \| \| w, wo \| with, without \| \| weight \| mass of … \| \| year \| from 01.01.2013 to day of sampling \| |
