# Supplementary material for: Habitat properties are key drivers of Borrelia burgdorferi (s.l.) prevalence in Ixodes ricinus populations of deciduous forest fragments
Source: Parasit Vectors. 2018 Jan 8;11:23. doi: 10.1186/s13071-017-2590-x (PMC5759830; doi:10.1186/s13071-017-2590-x)
Supplement: Supplementary file 6 — Details on the model building/variable selection procedure. (DOCX 12 kb) [file 13071_2017_2590_MOESM6_ESM.docx]

**Additional file 6: Text 3.**

## Model building/Variable selection

The overall statistical procedure is captured in Additional file 7: Figure S1. We used a supervised stepwise inclusion of variables: For all variables we calculated the model performance after adding the new variable to the existing model. Model performance was represented by AIC, the significance level of the variable in the resulting model and the coefficient of determination between the variable and the model without this variable (R²_V_) (to test “if the variable correlates with the model or actually its residuals”). We selected only variables with R²_V_ < 0.5 to avoid covariance. More significant variables were preferred over less significant variables, while variables were only considered if their p < 0.05. When significance levels and R²_V_ of several variables were similar, we chose the ecologically more meaningful and easier to interpret variable. We checked partial residual plots, marked data-points with an out-layer factor of more than 3 standard deviations from the mean thereof, plotted linear and polynomial trend lines for both, all data points and the subset of non-outliers and assured like that visually that a variable was not chosen based on influential outliers only. The value of AIC was constantly observed and it was assured that the overall model does not “drift away” (= the AIC became worse and worse with each iteration). We repeated this process until no variables fulfilling the above described characteristics remained. Questionable (i.e. ecologically hard to interpret) variables were kept out of the model and only included, if they repeatedly reemerged as “suitable variable”. We chose this hybrid procedure, because it enabled us to be in control of which variables are selected, assure that covariation is excluded and eventually come up with a model that makes sense ecologically, while at the same time not being limited to a handful of “expert-models”, which might not cover the full range of effects and hence lead to biased inference.
