# Supplementary figures and images for: Habitat properties are key drivers of Borrelia burgdorferi (s.l.) prevalence in Ixodes ricinus populations of deciduous forest fragments
Source: Parasit Vectors. 2018 Jan 8;11:23. doi: 10.1186/s13071-017-2590-x (PMC5759830; doi:10.1186/s13071-017-2590-x)

## Data Preparation

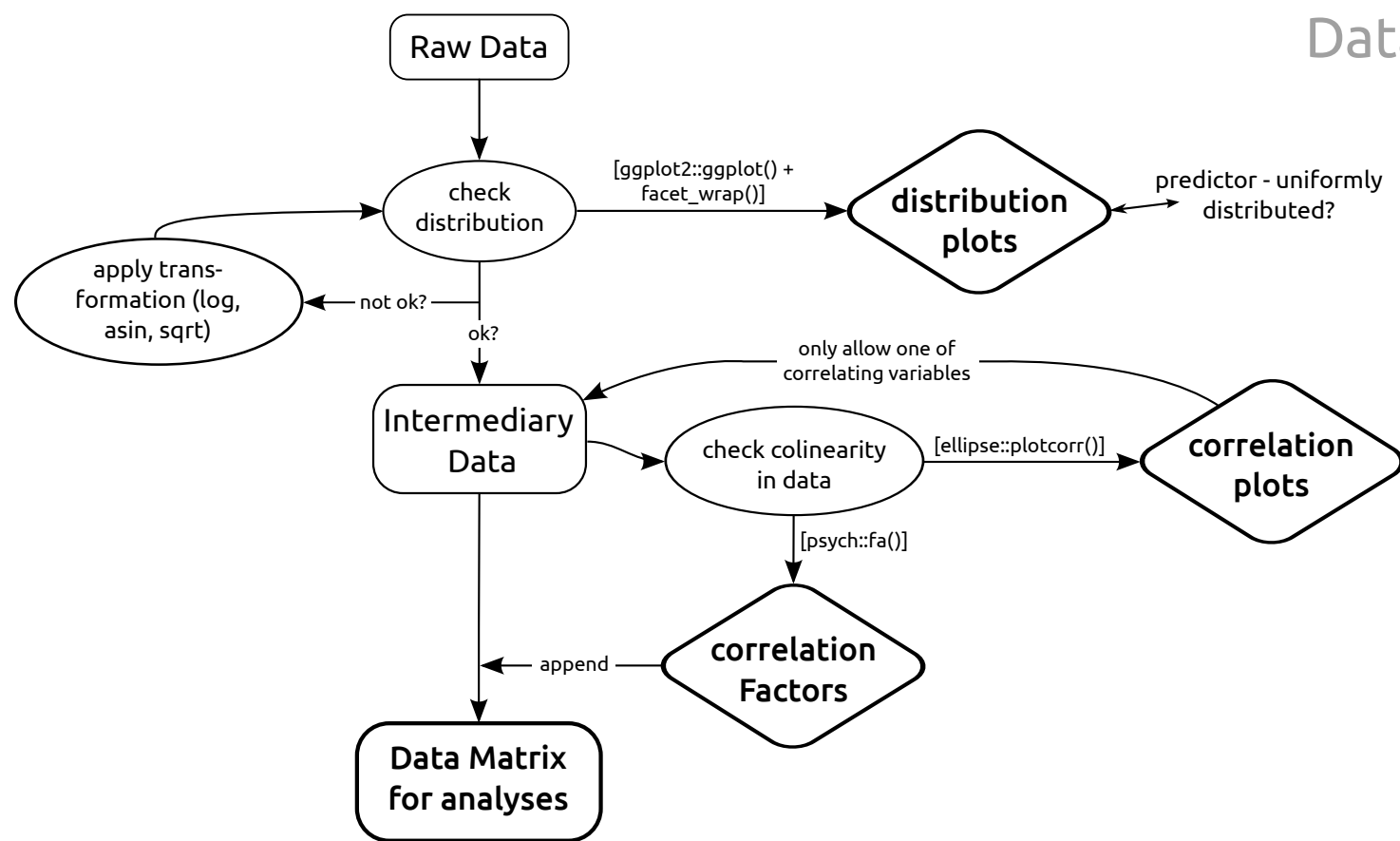

## Model Building

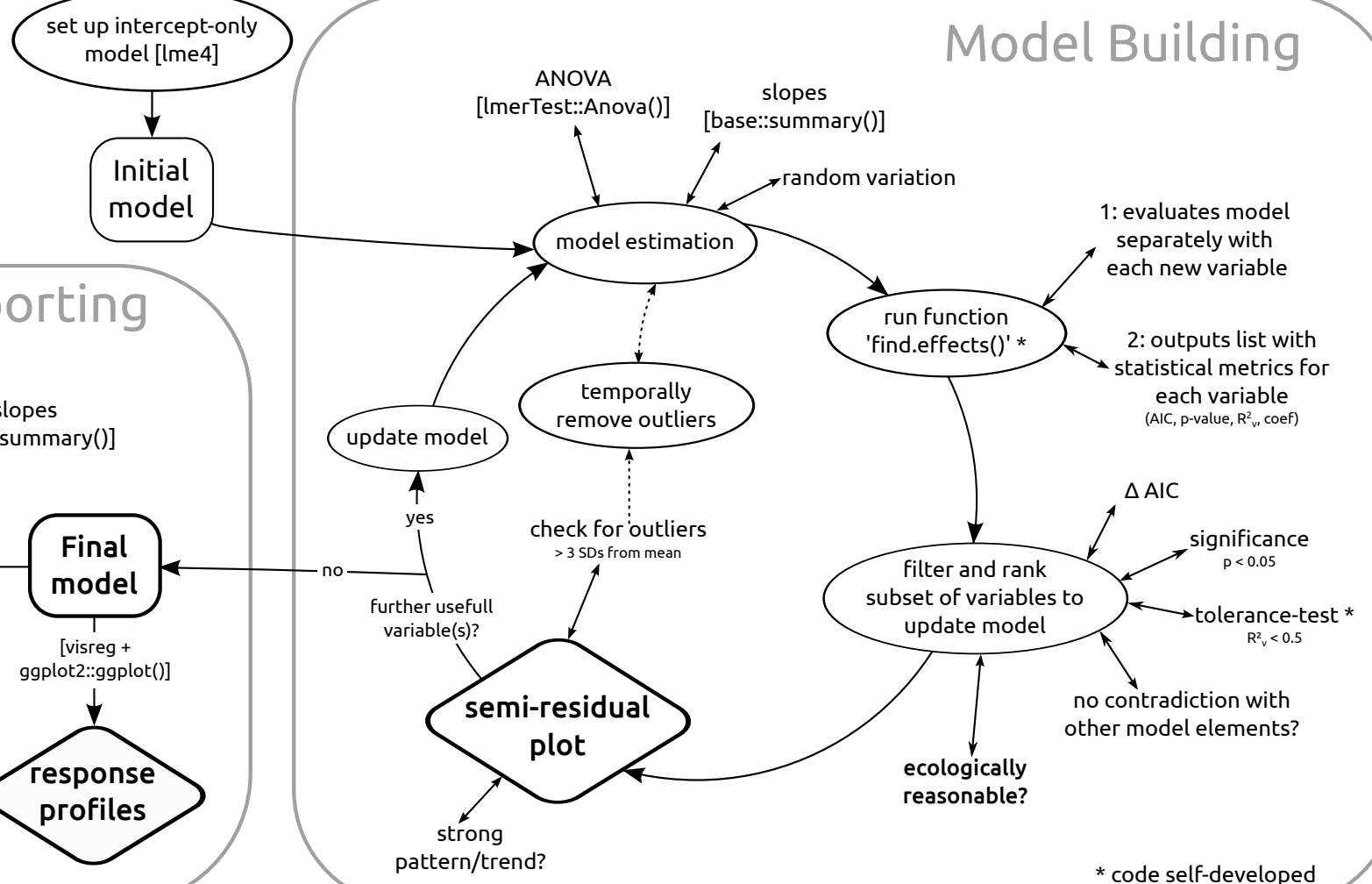

## Reporting

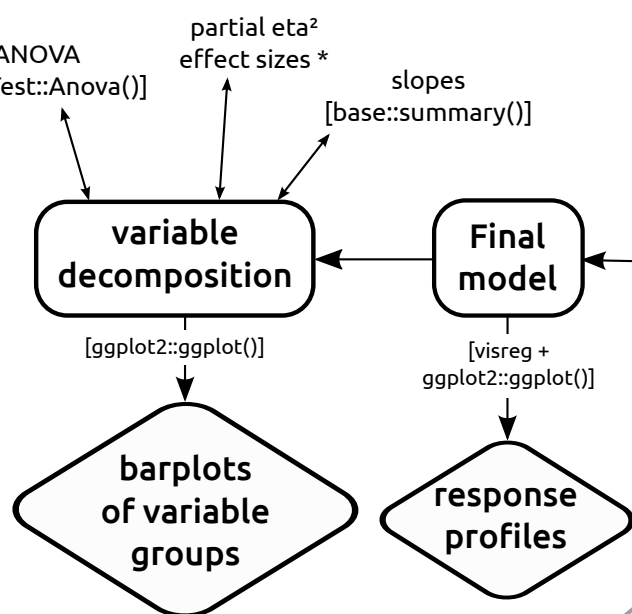

\* code self-developed

Supplement: Supplementary file 7 — Flow chart of the overall data processing procedure. (PDF 46 kb) [file 13071_2017_2590_MOESM7_ESM.pdf]
