# Supplementary material for: Habitat properties are key drivers of Borrelia burgdorferi (s.l.) prevalence in Ixodes ricinus populations of deciduous forest fragments
Source: Parasit Vectors. 2018 Jan 8;11:23. doi: 10.1186/s13071-017-2590-x (PMC5759830; doi:10.1186/s13071-017-2590-x)
Supplement: Supplementary file 10 — Response profiles of infection prevalence for each significant driver. Shown are the prediction line, confidence band (alpha = 0.05) and the partial residuals. The driver groups are macroclimate, landscape, macrohabitat, microhabitat and ontogeny. η2 values represent the relative contribution each variable has in explaining variation in the response. Nymphal infection prevalence. (FA) = variable is a correlation factor, ‘abund.’ = abundance, ‘disp.’ = dispersules, ‘cont.’ = content, CWM = community weighted mean, ‘reg. Leaf-dist.’ = leaf distribution regular on stem. (PDF 332 kb) [file 13071_2017_2590_MOESM10_ESM.pdf]

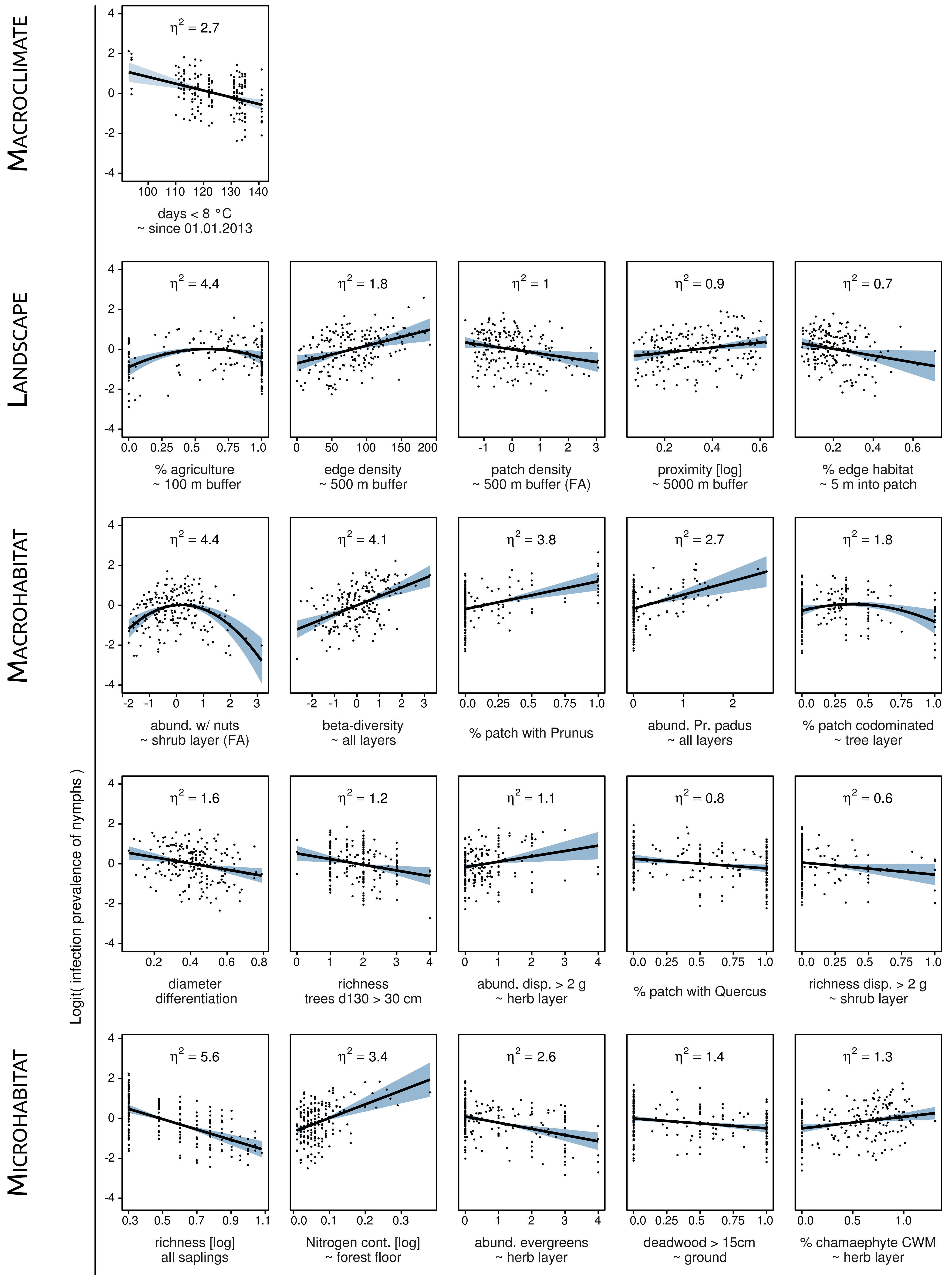

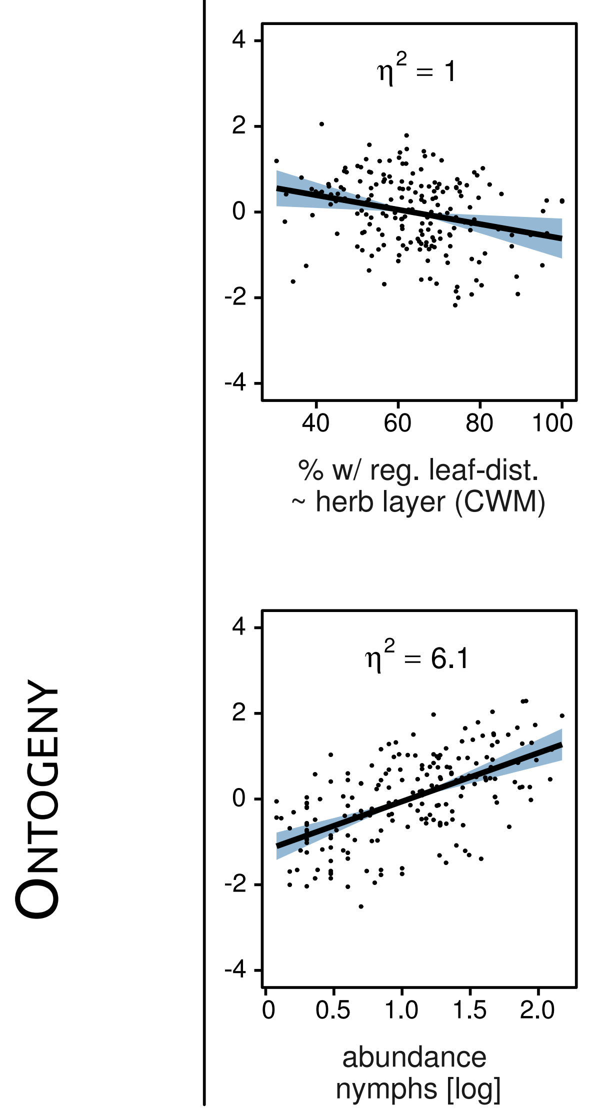

Additional file 10: Figure S2. Response profiles of infection prevalence for each significant driver. Shown are the prediction line, confidence band (alpha = 0.05) and the partial residuals. The driver groups are macroclimate, landscape, macrohabitat, microhabitat and ontogeny. η^2 values represent the relative contribution each variable has in explaining variation in the response. Nymphal infection prevalence. (FA) = variable is a correlation factor, 'abund.' = abundance, 'disp.' = dispersules, 'cont.' = content, CWM = community weighted mean, 'reg. leaf-dist.' = leaf distribution regular on stem.
