# Supplementary material for: Habitat properties are key drivers of Borrelia burgdorferi (s.l.) prevalence in Ixodes ricinus populations of deciduous forest fragments
Source: Parasit Vectors. 2018 Jan 8;11:23. doi: 10.1186/s13071-017-2590-x (PMC5759830; doi:10.1186/s13071-017-2590-x)
Supplement: Supplementary file 11 — Response profiles of infection prevalence for each significant driver. Shown are the prediction line, confidence band (alpha = 0.05) and the partial residuals. The driver groups are macroclimate, landscape, macrohabitat, microhabitat and ontogeny. η2 values represent the relative contribution each variable has in explaining variation in the response. Adult infection prevalence. (FA) = variable is a correlation factor, ‘abund.’ = abundance, ‘disp.’ = dispersules, ‘SLA’ = specific leaf area, ‘tree2’ = lower tree layer. (PDF 254 kb) [file 13071_2017_2590_MOESM11_ESM.pdf]

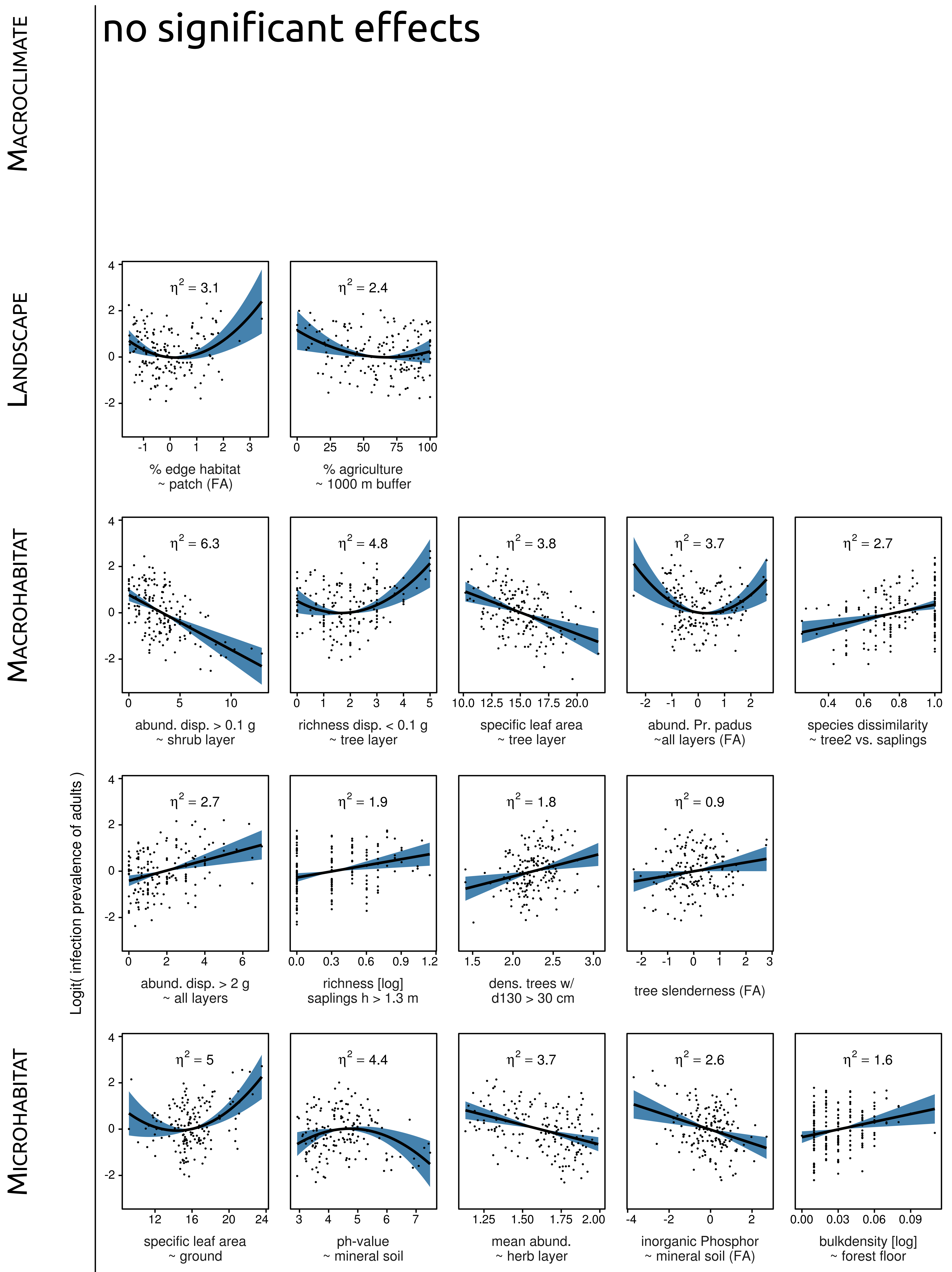

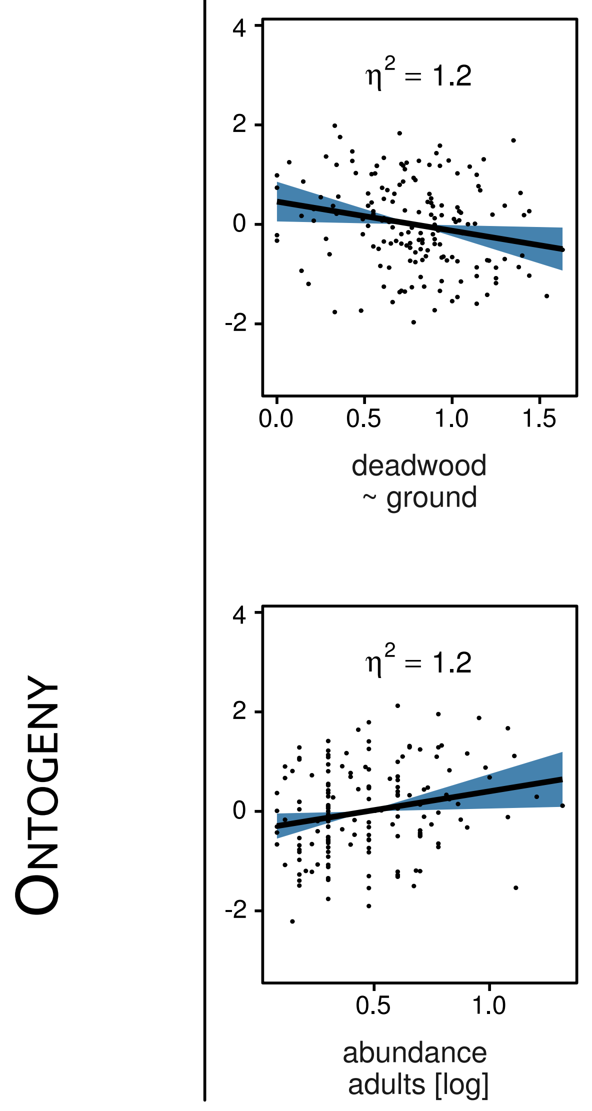

Additional file 11: Figure S3. Response profiles of infection prevalence for each significant driver. Shown are the prediction line, confidence band (alpha = 0.05) and the partial residuals. The driver groups are macroclimate, landscape, macrohabitat, microhabitat and ontogeny. η² values represent the relative contribution each variable has in explaining variation in the response. Adult infection prevalence. (FA) = variable is a correlation factor, 'abund.' = abundance, 'disp.' = dispersules, 'SLA' = specific leaf area, 'tree2' = lower tree layer.
